# LncRNA RPL29P2 promotes peritoneal fibrosis and impairs peritoneal transport function via miR-1184 in peritoneal dialysis

**DOI:** 10.7150/ijms.93547

**Published:** 2024-04-15

**Authors:** Huan Li, Yuting Zhang, Mingwen Che, Hanmin Wang, Sutong Li, Peng He, Shiren Sun, Guoshuang Xu, Chen Huang, Xiaowei Liu, Ming Bai, Meilan Zhou, Binxiao Su, Peng Zhang, Lijie He

**Affiliations:** 1 Department of Nephrology, Xijing Hospital, the Fourth Military Medical University, Xi'an, Shaanxi, China.; 2 Department of Nephrology, the Second People's Hospital of Shaan xi Province, Xi'an, Shaanxi, China.; 3 Department of Medicine, the 951 Hospital of the PLA, Korla, Xinjiang, China.; 4 Department of Nephrology, Xi'an Central Hospital, Xi'an, Shaanxi, China.; 5 Department of Anesthesiology and Perioperative Medicine, Department of Intensive Care Unit, Xijing Hospital, Fourth Military Medical University, Xi'an, Shaanxi 710032, China.

**Keywords:** LncRNA RPL29P2, miR-1184, collagen, peritoneal dialysis, fibrosis, peritoneal transport function

## Abstract

Peritoneal dialysis (PD), hemodialysis and kidney transplantation are the three therapies to treat uremia. However, PD is discontinued for peritoneal membrane fibrosis (PMF) and loss of peritoneal transport function (PTF) due to damage from high concentrations of glucose in PD fluids (PDFs). The mechanism behind PMF is unclear, and there are no available biomarkers for the evaluation of PMF and PTF. Using microarray screening, we found that a new long noncoding RNA (lncRNA), RPL29P2, was upregulated in the PM (peritoneal membrane) of long-term PD patients, and its expression level was correlated with PMF severity and the PTF loss. *In vitro* and rat model assays suggested that lncRNA RPL29P2 targets miR-1184 and induces the expression of collagen type I alpha 1 chain (COL1A1). Silencing RPL29P2 in the PD rat model might suppress the HG-induced phenotypic transition of Human peritoneal mesothelial cells (HPMCs), alleviate HG-induced fibrosis and prevent the loss of PTF. Overall, our findings revealed that lncRNA RPL29P2, which targets miR-1184 and collagen, may represent a useful marker and therapeutic target of PMF in PD patients.

## Introduction

Peritoneal dialysis (PD) is a renal replacement therapy which might encompasse different side effects including peritoneal membrane fibrosis (PMF) with changes in morphology and function in the peritoneal membrane (PM) and loss of peritoneal transport function (PTF) occurring due to damage caused by high glucose stimulation in PD fluids (PDFs) 1, 2. During the first two years of PD treatment, there is no apparent structural damage in the PMs of the patients, except for epithelial-to-mesenchymal transition (EMT), cell morphology, cytoskeletal reorganization, and decreased adhesion and movement in human peritoneal mesothelial cells (HPMCs) 1, 3, 4. High glucose (HG) has been suggested to induce the epithelial-mesenchymal trans-differentiation of the peritoneal mesothelial cells via multitudinous modulation of molecular pathways, finally causing cytoskeletal protein reorganization, decreased adhesive ability, and production of more extracellular matrix (ECM)^5^-^8^. However, the mechanism behind these changes remains unclear, and there are no available biomarkers for the evaluations of PMF and PTF loss. Our previous work revealed that serum response factor (SRF) was involved in the phenotypic transition and fibrosis of the PM through direct regulation of microRNAs (miRNAs) or miRNA clusters 9, 10. Now, more stable and easily detected biomarkers in HPMCs after HG damage had been discovered.

Recent studies proved that lncRNAs could participate in many kinds of biological processes, as seen in cancer metastasis, and the fibrosis of kidney, lung, and heart 11-14. For example, lncRNA H19 was overexpressed in kidney development after high glucose stimulation and contribute kidney fibrosis progression 13. And in endothelial cells, lncRNA Malat1 was found that it could lead to hyperglycemia-induced microinflammation and take part in the initiation of microglial pyroptosis and neuronal death in retinal ischemia/reperfusion injury 13. Some study showed that in liver fibrosis, down-regulating H19 of macrophages could depress the macrophage polarization and recruitment, and suppress cholangiocyte proliferation, cholestatic liver injuries and fibrosis 15. Other studies in diabetic nephropathy showed that lncRNA SOX2OT was markedly down-regulated in DN mice and the mesangial cells in kidney. Up-regulating of lncRNA SOX2OT could alleviate DN-induced renal injury diminish by inhibition of Akt/mTOR and suppressing of autophagy 16. Another pathway, Kcnq1ot1/miR-214-3p/caspase-1/TGF-β1 could also alleviate pyroptosis and cardiac muscle cell fibrosis in diabetic cardiomyopathy 17. Knockdown of XIST could inhibit renal tubular epithelial cells apoptosis, inflammation and renal fibrosis by TGF-β1 and the down-regulating the pathway of miR-19b-SOX6 in UUO mice 18. All these studies highlighted the significance of lncRNAs as regulatory molecules in heart fibrosis, cardiac autophagy, hypertension, acute kidney injury, glomerular diseases, acute allograft rejection, and renal cell carcinoma 19-21. However, the role of lncRNAs in peritoneal fibrosis needs further elucidation. Therefore, in the present work, we investigated the role of a LncRNA, RPL29P2, in peritoneal membrane fibrosis both *in vitro* and in PD animal model.

## Materials and Methods

### Ethical Statement

The current work was strictly conducted incompliance with the guidelines of the 1957 Declaration of Helsinki for the use of laboratory animals. The trial was approved by the ethics committee of the animal experiment center of the Fourth Military Medical University (permit number KY20173279-1) and was approved by the ethics committee of the Clinical Trial Ethics Committee of Xijing Hospital, Fourth Military Medical University (permit number KY20183103-1).

### Patient Selection

PD patients were included in our peritoneal dialysis center for examination and follow-up starting from December 1, 2017. All the participants started PD as renal replacement therapy. This was followed by regular follow-up. Exclusion criteria were as follows: (A) combined with recent active infection, malignancies, liver diseases, hematological diseases or active autoimmune diseases; (B) confirmed peritonitis caused by the combination of other diseases (such as tuberculous peritonitis, drug-induced retroperitoneal fibrosis); (C) younger than 18 years old; (D) regular PD for less than 36 months; (E) patients with a history of previous hemodialysis (HD), or kidney transplant; (F) without obtaining informed consent. According to the optimal selection criteria, three PD patients were ultimately included. Starting from the first peritoneal dialysis, collected and analyzed the peritoneal effluents from the PD patients for 6, 18 and 36 months. The control group selected ESRD (end-stage renal disease) patients who received catheter insertion therapy for the first time and matched the gender and age of the PD group.

### Animals and Experimental procedures

8-week old Female SD rats (weighing 220±20 g) were selected purchased from the animal center of Fourth Military Medical University and were kept under specific pathogen-free (SPF) conditions with food and water *ad libitum*. Then the chronic infusion rat model of high-glucose induced PD fibrosis rats will be made.

Experimental procedures were conducted for 6 weeks. SD rats were randomly divided into 5 groups: the control (no intervention), NaCl (100ml/kg/d), mannitol, HG-PDF (4.25% glucose dialysis solution, 100ml/kg/d), and AAV (injection of virus on day 15) groups. Subjects in the control, NaCl and Mannitol groups were intraperitoneally injected with a volume of 25 ml twice daily for 6 weeks. Animals in the PD group were injected continuously for 3 days starting on the second week, with a daily dose of 4.25% PD solution containing 0.5% lipopolysaccharide (50 ml) to promote peritoneal fibrosis 22. Rats in the AAV group were injected with Adeno-associated virus (AAV) after 2 weeks, and each rat was intraperitoneally injected with 2×10^10^ viral genomes. The virus sequence design and synthesis were completed by Shanghai GeneChem Co., Ltd., Shanghai, China. (Table S4).

The severity of PF is mainly determined based on two aspects. One is the thickness of the peritoneum. The second is the immunohistochemical staining results of fibrosis related molecules in peritoneal tissue, specifically referring to the expression of molecules such as SMA, fibronectin (FN), collagen and E-cadherin (E-cad).

PTF was measured through peritoneal equilibration test (PET). At 4 hours, the dialysate-to-plasma ratio of creatinine (D/P Cr) and the initial dialysate-to-end dialysate ratio of glucose (D/D_0_ glucose) were measured. According to peritoneal transport function, it is sequentially divided into and low transport (L), low average transport (LA), high transport (H) and high average transport (HA)^23^. When PTF of PD patients transitions from L to H, it indicates a decrease in PTF.

### Microarray analysis

Proceed in the following order: 1. RNA quantification and qualification; 2. Library preparation for Transcriptome sequencing; 3. Clustering and sequencing (Novogene Experimental Department).

Data analysis: (1). Raw data (raw reads) of FASTQ format were firstly processed through in-house Perl scripts; (2). Reads mapping to the reference genome. Reference genome and gene model annotation files were downloaded from genome website directly. Index of the reference genome was built using Hisat2 v2.1.0 and paired-end clean reads were aligned to the reference genome using Hisat2 v2.1.0; (3). Quantification of gene expression level. FeatureCounts v1.6.4 was used to count the reads numbers mapped to each gene. And then FPKM of each gene was calculated based on the length of the gene and reads count mapped to this gene; (4). Differential expression analysis. Differential expression analysis of two conditions/groups (two biological replicates per condition) was performed using the DESeq2 R package (1.24.0); (5). Gene Ontology (GO) and KEGG enrichment analysis of differentially expressed genes. GO enrichment analysis of differentially expressed genes was implemented by the clusterProfiler R package (3.12.0), in which gene length bias was corrected. GO terms with corrected *P* value < 0.05 were considered significantly enriched by differential expressed genes.

### Peritoneal immunohistochemistry

At the end of the sixth week, animals were euthanized by CO_2_ asphyxiation and peritoneal membranes were collected from the different experimental groups. Collected tissues were embedded in paraffin and sectioned at 5 µm using microtome. Paraffin sections were soaked in xylene and dehydrated in ethanol followed by antigen retrieval protocol. Then, the tissue sections were soaked in 200 ml of methanol and 2 ml of 30% H_2_O_2_ for 15 min and blocked with normal goat serum for 30 min, slices were then incubated with the primary antibodies in Phosphate-buffered saline (PBS) (1:100 dilution) at 4 °C overnight. Then tissue slices were incubated with the secondary antibody of the corresponding species (rat/rabbit), and the sections were incubated in streptomycin ovalbumin working solution and stained with DAB as previously described 10. Images were captured with microscope (Olympus, BX55, Germany) and analyzed using image IM50 software (Leica, Wetzlar, Germany).

### Cell culture and tissue collection

The immortal HPMC lines were cultured in DMEM medium with 10% FBS, which was not altered. Primary human mesothelial cells were collected from PD patient effluents of Xijing Hospital. The inclusion criteria were as follows: age less than 65 years, the duration of CAPD treatment longer than 1 month, no history of peritonitis in the last 6 months, use of dialysis solution with 1.5% glucose, and no history of abdominal surgery or immunosuppressant use in the last 6 months (Table S4). Patients were sorted into five groups (G indicates “group”) by dialysis time. G0 comprised 5 ESRD patients who underwent catheterization surgery and served as the control group. G1 comprised patients with 1 to 6 months of dialysis, G2 comprised those with 7 to 24 months of dialysis, G3 comprised those with 25 to 48 months of dialysis, and G4 comprised those treated over 48 months of dialysis (Table S4).

### Cell transfection

MiR-1184 inhibitor or mimic were acquired from GeneChem company (Shanghai, China). MiR-1184 mimic and inhibitor oligos were designed and cloned into the pGCSIL-GFP vector. HPMCs were transfected with lentivirus containing 10 mg/ml polybrene, and the empty lentivirus was used as a control. The transfected HPMCs either with the miR-1184 mimic or inhibitor vectors were collected after 48 or 72 h.

### Cell immunofluorescence staining

Cells were fixed with 4% paraformaldehyde for 20 min at room temperature, lysed at 4 °C in prechilled 0.1% Triton X-100, and then incubated for 1 h with IH blocking solution and primary antibodies at 4 °C overnight (dilution ratios: COL1A1, 1:200; E-cad, 1:200; α-SMA, 1:200, santa cruze Biotechnology, CA, USA). Then, the cells were incubated with a fluorescent secondary antibody (FITC-conjugated, 1:200 dilution, Invitrogen, CA, USA) and stained with DAPI. Slides were sealed with a fluorescence decay resistance sealant and stored at -20 °C.

### Western Blotting (WB)

The bottom surface of the cultured dishes was washed with prechilled (4 °C) PBS, and the cells were then scraped and centrifuged. Pelleted cells were mixed with RIPA buffer containing protease inhibitors and incubated on ice. Protein extract was separated by SDS‒PAGE (Boster Biological Technology, Wuhan, China) and transferred to polyvinylidene fluoride (PVDF) film (millpore, USA). The PVDF film was removed and marked with a ballpoint pen, and the target strips were cut. Then, the strips were incubated in 10% skim milk powder for 1-2 h and incubated with antibody diluted in accordance with the product manual at 4 °C overnight. The antibodies were used as before, and the dilution ratios were COL1A1, 1:200; E-cad, 1:150; α-SMA, 1:200, CTGF: 1:150, SRF 1:200, vim 1:200, and β-actin: 1:1000 (santa cruze Biotechnology, CA, USA). Then, the strips were incubated with diluted rabbit/mouse secondary antibody (Boster Biological Technology, Wuhan, China) and visualized in a developer using chemiluminescence reagents. The gray values of the bands on the strips were measured with Amersham Imager 600 software.

### RNA extraction and quantitative PCR (qPCR)

Total RNA was extracted from HPMCs or renal tissues by RNAiso Plus buffer (total RNA extraction reagent, Takara, Japan). Total RNA was reverse transcribed to cDNA. PCR amplification was performed using a PCR system (Applied Biosystems) under the following cycling conditions: 30 cycles (of 98 °C for 10 s, 55 °C for 5 s, and 72 °C for 5 s). The reaction was loaded onto a 1% agarose gel with a DNA molecular ladder ranging from 0.1 to 10 kilobase pairs (kbp). Relative quantification of mRNA expression was performed via the 2-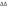
CT method with normalization to β-actin as housekeeping gene. MiR-1184 expression was normalized to U6 as the internal control. Real-time PCR primer sequences are shown in Table S5.

### Fluorescence *In Situ* Hybridization (FISH)

The concentrated probe was incubated at 75 °C for 5 min and immediately placed on ice to separate the two strands of the probe (Table S5). The tissue biopsy was incubated at 37 °C for 1 h on a baking sheet, soaked in 2× SSC at 37 °C for 2 min, added to proteinase K digestion for 30 min and washed in 2× SSC. Dehydration was performed in prechilled (-20 °C) 70%, 85%, and 100% ethanol for 5 min each, and the dehydrated biopsy tissue was washed with 2× SSC at 37 °C. Hybridization buffer containing the probe was added dropwise to the cover glass after enzymatic inactivation and incubated in the dark in a wet box at 37 °C overnight. Then, the biopsy tissue was washed in 2× SSC containing 70% deionized formamide, preheated to 43 °C, washed in 2× SSC and stained with DAPI. A fluorescence antifade agent was used to seal the tissue biopsy and stored at -80 °C in the dark.

### Primary cell culture and staining

Transabdominal fluid was centrifuged at 800 r/min for 5 min. The cell precipitate was cultured in DMEM+10% FBS, the medium was replaced after 24 h, and the HPMCs ex vivo were fixed with 4% paraformaldehyde after 48 h. FISH was performed as previously described.

### Chromatin immunoprecipitation

Chromatin immunoprecipitation (ChIP) was performed as previously described. These collected fragments or oligos were coimmunoprecipitated with antibody or rabbit IgG as a control. Total RNA was extracted using a RNeasy Kit (Qiagen, Germany). The purified DNA oligos were made as templates. Quantitative PCR was carried out by an ABI 7900 Sequence Detection System and SYBR Green PCR Core Reagent Kit (Life Technologies company). The designed primers are shown in Supplemental Table S4.

### Reporter vector construction and luciferase assay

The promoter of RPL29P2 with the CArG element was amplified by PCR from the HPMCs. The PCR products were ligated (Takara, Japan) into the pRL-SV40 vector (Promega, USA). The HPMCs were transfected with OPTI-MEM reagent (Invitrogen, USA). The transfections were repeated three times. The extraction was prepared for the luciferase assay (Promega) after transfection by an Assay Kit (Promega, USA) (Fig. 3).

The 3'-UTR fragments of RPL29P2 or COL1A1 were amplified by PCR from the HPMCs. The products were gel-purified and ligated (Takara, Japan) into the psiCheck-2 plasmid (Promega, USA), named psi-CHECKTM-2- RPL29P2 or COL1A1. The mutated sequences were constructed by a KOD-plus Mutagenesis Kit (). HG- HPMCs were cotransfected with miR-1184 (1 µl, 20 µM), wild-type (wt, 0.5 µg) or mutant (mut, 0.5 µg) RPL29P2 or COL1A1 vector and Lipofectamine 2000 (1 μl, Invitrogen, USA). Luciferase activity was tested using the Dual-Glo luciferase assay kit (Promega, USA).

### Statistical analyses

Each experiment was performed in triplicate. The densities of the bands from WB and the PCR data were quantified using Quantity One software (Bio-Rad, Hercules, CA). Data are presented as the means±SEM. Differences between the means were evaluated using a 2-tailed unpaired Student's t test or one-way ANOVA. The statistical analyses were performed using SPSS statistics software, version 22.0 (IBM, Inc., Chicago, IL, USA) and GraphPad Prism software, version 8.0 (La Jolla, CA, USA). All hypothesis tests were two-sided, and *P* < 0.05 was considered statistically significant.

## Results

### LncRNA RPL29P2 was upregulated in PM tissues of long-term PD patients and related to PMF and the loss of PTF

**Figure 1:**
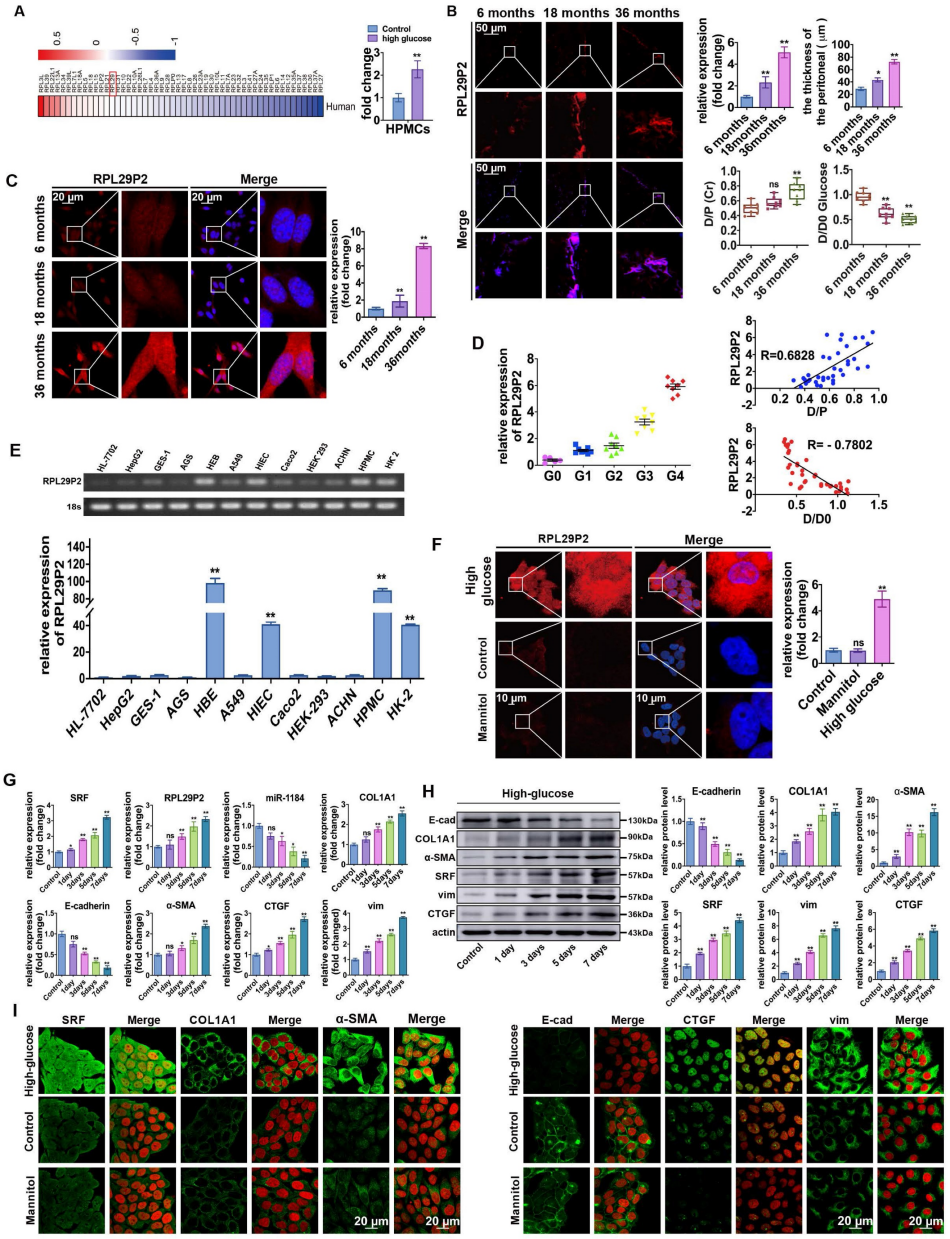
** Upregulation of lncRNA RPL29P2 in the phenotypic transition of HG-stimulated HPMCs *in vitro* and *in vivo*. (A)** Heatmap showing lncRNA expression changes that were twofold in HPMCs cultured in HG medium for 72 h. PCR was used to confirm the array results.** (B)** RPL29P2 was detected by FISH in the peritoneal tissues of PD patients who were treated for 6, 18 and 36 months. The peritoneal thickness of each group was measured. The dialysate-to-plasma ratio of creatinine (D/P Cr) and the initial dialysate-to-end dialysate ratio of glucose (D/D_0_ glucose) were used to evaluate the transport function of the PM. ^**^P<0.05 versus the patient with 6 m PD treatment. **(C)** RPL29P2 was detected by FISH in effluent-derived HPMCs of the three patients. **(D)** qPCR showed the expression of RPL29P2 in effluent-derived HPMCs from 32 PD patients (G1-G4) who underwent PD treatment compared to the expression in omentum-derived HPMCs from five patients with ESRD who underwent catheterization surgery for further PD therapy as a control group (G0). Correlation analysis showed that D/P and RPL29P2 and D/D_0_ and RPL29P2 in effluent-derived HPMCs from groups G0 to G4. R Sq Line ar, R-squared line-adjusted rate. **(E)** qPCR showed the expression of lncRNA RPL29P2 in HL-7702, HepG2, GES-1, AGS, HBE, A549, HIEC, Caco2, HEK239T, ACHN, HPMCs, and HK-2 cells. **(F)** The expression of RPL29P2 was detected by FISH in HPMCs stimulated with 60 mmol/L HG for 72 h and was compared to that in control cells and mannitol-treated cells. **(G, H, I) q**PCR, WB analysis and immunofluorescence staining showed the expression of SRF, RPL29P2, miR-1184, COL1A1, E-cad, α-SMA, CTGF, and vimentin in HPMCs stimulated with 60 mmol/L HG for 0 days, 1 day, 3 days, 5 days, and 7 days. ns, not significant; *P<0.05; **P<0.01.

Through microarray profiling (Fig. 1A, Table S1), 12 genes in the RPL family were found to be increased in HG-stimulated HPMCs. Furthermore, qPCR results showed that RPL29P2, from the RPL family, was significantly upregulated in HG-stimulated HPMCs compared to mannitol-stimulated control cells. RPL29P2 is located on chromosome 17 in humans, and a clear ortholog named Rpl29 was identified in the rat genome via Basic Local Alignment Search Tool (BLAST) analysis, which is 80% homologous with human RPL29P2. Therefore, we selected the lncRNA RPL29P2 for further experiments.

The expression of RPL29P2 in PM tissues and cast-off HPMCs from peritoneal effluents in three PD patients who were treated with PD for 6, 18 and 36 months was evaluated (Fig. 1B, 1C and Table S2A). Fluorescence *in situ* hybridization (FISH) demonstrated that RPL29P2 was weakly expressed in the PM tissues of short-term PD patients (6 months) compared to the PM tissues of long-term PD patients (18 and 36 months). The PTF of the three patients, the dialysate-to-plasma ratio of creatinine (D/P Cr) and the initial dialysate-to-end dialysate ratio of (D/D_0_) glucose at 240 minutes, were surveyed using a peritoneal equilibration test. The D/P (Cr) was significantly higher at 36 months; while the D/D_0_ values were significantly lower at 18 and 36 months. Moreover, the thickness of the peritoneal membrane was the highest at 36 months (*P* < 0.01). In primary cast-off HPMCs ex vivo, FISH demonstrated that RPL29P2 was upregulated and strongly expressed in the nucleus and cytoplasm of HPMCs in the long-term patients (18 and 36 months) compared to short-term (Fig. 1C). We investigated the levels of RPL29P2 in more sloughed HPMCs ex vivo from 32 PD patients by PCR (Fig. 1D, Table S3). According to the CAPD time, we divided the patients into five groups (G0, G1, G2, G3, and G4). This result suggested that RPL29P2 gene expression was positively correlated with D/P Cr but a negative correlation to D/D_0_ glucose in PD patients was observed. RPL29P2, as determined by PCR, was expressed at lower levels in human liver and gastric cells and in the corresponding tumor cells than that in other tissues (Fig. 1E). Moreover, RPL29P2 was significantly upregulated in HPMCs and HK-2 cells but exhibited lower expression in these cancer cells.

FISH showed that RPL29P2 was localized primarily in the cytoplasm and nucleus of immortal HPMCs after HG stimulation compared to its localization in control (no intervention) or mannitol-treated HPMCs *in vitro* (Fig. 1F). qPCR analysis confirmed that RPL29P2 was constitutively expressed in HPMCs and was upregulated after HG stimulation (Fig. 1G) compared to mannitol-treated HPMCs (Fig. S1A). In addition, the mRNA expression of RPL29P2 in response to HG was time-dependent. qPCR, WB and IH showed the significant upregulation of the marker proteins alpha-1 type I collagen (COL1A1), SRF, vimentin (vim), alpha smooth muscle actin (α-SMA) and connective tissue growth factor (CTGF), while the significant downregulation of E-cadherin (E-cad) in HG-stimulated HPMCs (Fig. 1H, 1I Fig. S1B) was observed.

### LncRNA RPL29P2 was highly expressed in HG-stimulated PD rats and attenuated by CCG-1423

**Figure 2:**
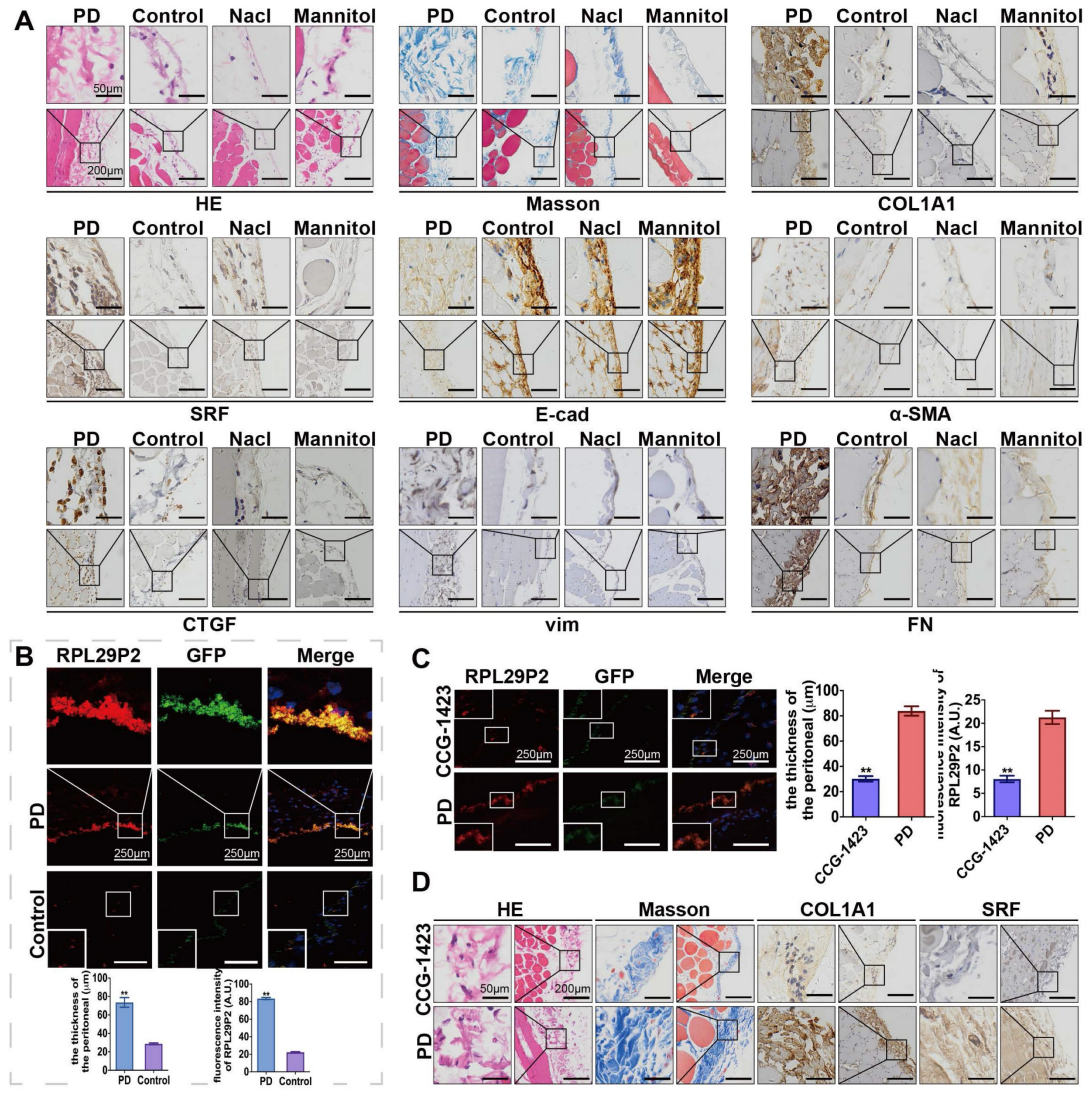
**RPL29P2 was highly expressed in PD rats and attenuated in PD rats treated with the SRF inhibitor CCG-1423. (A)** H&E, Masson and IH staining showing the thickness of PMs in PD rats treated with HG-PD fluid and the expression of SRF, COL1A1, E-cad, α-SMA, CTGF, vimentin and FN in peritoneal tissues. **(B)** FISH showing RPL29P2 expression in the peritoneal tissues of PD rats compared with the control (no treatment), NaCl and mannitol groups. **P<0.01. **(C)** The expression of RPL29P2 in peritoneal tissues of PD rats after treatment with CCG-1423. **P<0.01. **(D)** The expression of COL1A1 and SRF in PD rats treated with CCG-1423.

A PD animal model was established by intraperitoneal injection with HG-PD fluid (HG-PDF) (4.25% glucose dialysis solution, Baxter, USA) or 0.9% saline solution for 6 weeks (8 rats per group). The tissue fluorescence in adeno-associated virus (AAV)-control rats showed a thicker PM in the PD rats (84.08±7.77 μm) compared to the control (23.63±5.76 μm), saline (26.58±4.23 μm), and mannitol (24.82±5.98 μm) groups (Fig. 2A-B, Fig. S2A-C, Table S2B). In addition, hematoxylin and eosin (H&E), Masson and immunohistochemistry (IH) staining showed a thicker PM; higher expression of SRF, COL1A1, α-SMA, CTGF, vimentin (vim), and fibronectin (FN); and lower expression of E-cadherin (E-cad) in PD model rats than in control rats (no intervention). Analysis of RPL29P2 by FISH showed that RPL29P2 was upregulated in the PM of HG-PDF rats, which exhibited a thicker PM than the control rats.

We injected CCG-1423 (SRF inhibitor) into the PD rat model (Fig. 2C-D, Table S2D) to inhibit the expression of SRF. A thicker PM and loss of RPL29P2 expression were found in the parietal peritoneum in the CCG-1423 group (30.15±3.77 μm) than in the PD group (83.87±6.48 μm), as detected by FISH, H&E and Masson staining.

### The transcription factor SRF directly promoted the expression of RPL29P2, which could inhibit the phenotypic transition via miR-1184 and collagen

**Figure 3:**
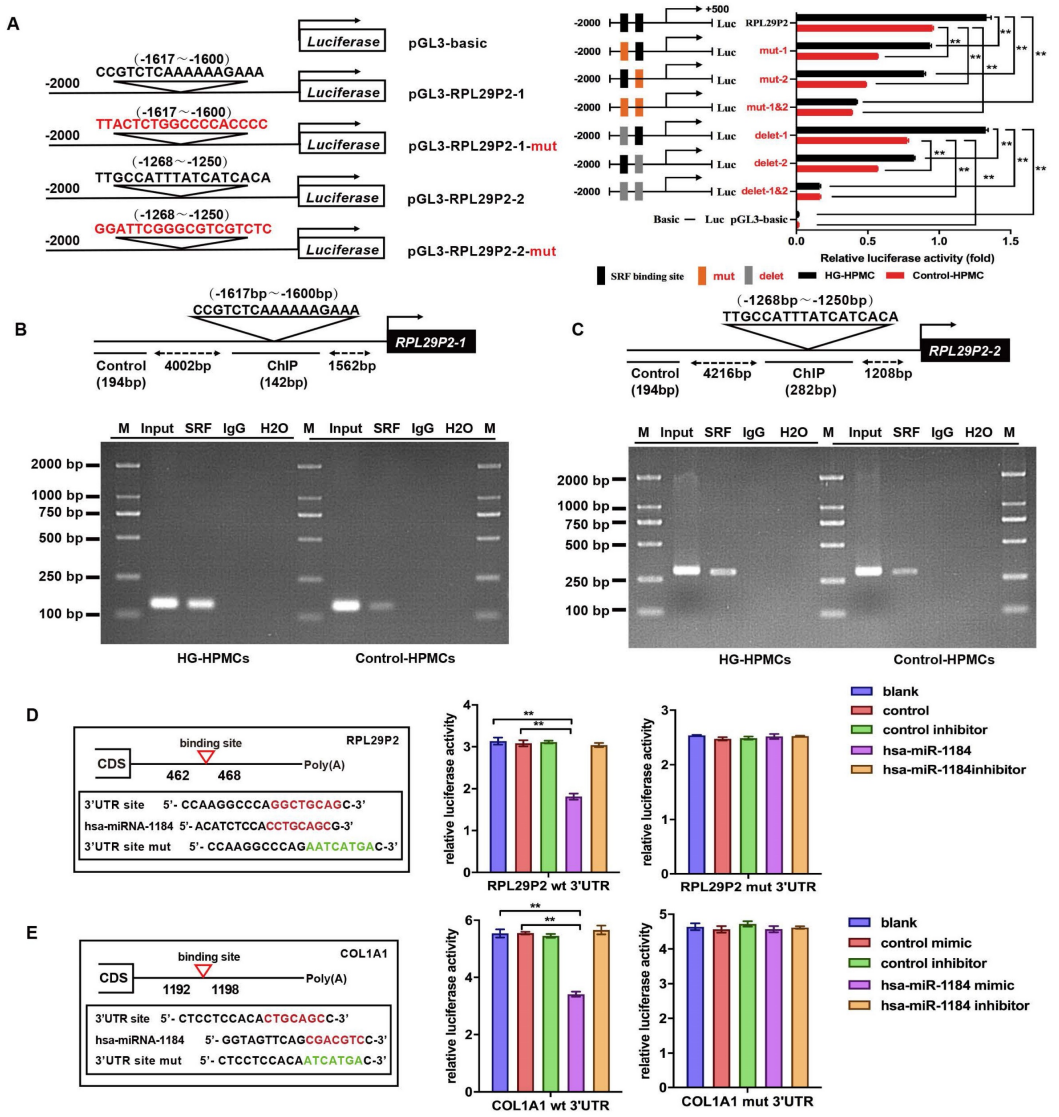
** RPL29P2 was directly promoted by SRF and inhibited EMT in HPMCs via miR-1184 and COL1A1. (A-C)** ChIP analysis showed the association between RPL29P2 and SRF in HG-stimulated HPMCs compared with control HPMCs. Endogenous SRF protein in HG-stimulated HPMCs was immunoprecipitated using an anti-SRF antibody and subjected to PCR analysis with RPL29P2 primers. The conserved upstream region of the RPL29P2 gene was cloned into the pGL3 luciferase expression plasmid and transfected into control HPMCs or HG-stimulated HPMCs. ** *P*<0.01. luc, luciferase. **(D, E)** The luciferase reporter gene array assay confirmed the miR-1184 target RPL29P2 or COL1A1 in HPMCs. Predicted duplex formation between the RPL29P2 and COL1A1 3'-UTRs and the miR-1184 binding sites within the wild-type (wt) RPL29P2 and COL1A1 3'-UTRs and the mutant (mut) RPL29P2 and COL1A1 3'-UTRs. Luciferase activity of wt and mut RPL29P2 or COL1A1 3' UTR reporter genes in HPMCs transfected with the miR-1184 mimic or inhibitor compared with HPMCs transfected with empty mimic or inhibitor oligonucleotides, respectively. ** *P*<0.01. CDS, coding sequence; NC, nonspecific control; ns, not significant; UTR, untranslated region.

We found that SRF was overexpressed after HG induction in a time- and dose-dependent manners. Two predicted SRE sites (CArG elements), from -1617 to -1600 and -1268 to -1250, were found in the promoter of RPL29P2 on chromosome 17; these sites could bind to the SRF protein (Fig. 3A-C). Strong luciferase was detected in HG-treated HPMCs harboring the wild-type 5' UTR in RPL29P2. Furthermore, luciferase activity was higher in HG-stimulated HPMCs than in control HPMCs. However, RPL29P2 was suppressed by CArG box mutation or deletion. Thus, these results showed that SRF could bind to the two promoter regions in the RPL29P2 gene cluster and promote its transcription.

We then analyzed potential downstream miRNAs of RPL29P2 using prediction algorithms (Fig. 3D). Among these predicted target miRNAs, miRNA-1184, which has a decreased expression in HG-stimulated HPMCs, and COL1A1, as its predicted target, might be a target of RPL29P2. We predicted the binding site of RPL29P2 in miR-1184 and wild-type and mutant fragments of the RPL29P2 mRNA 3' UTR from positions 462 to 468 (RPL29P2 wt 3' UTR and RPL29P2 mut 3' UTR, respectively) and inserted them into the region of the reporter gene. Next, hsa-miR-1184 mimic or inhibitor oligos were transfected with the 3' UTR luciferase complex into HG-stimulated HPMCs. We found that miR-1184 weakened the luciferase activity of the wild-type RPL29P2 3' UTR, but the mutant RPL29P2 3' UTR was unchanged.

COL1A1, which participates in encoding collagen proteins, is involved in ECM deposition and might be one of the downstream targets of miR-1184 (Fig. 3E). To determine whether COL1A1 is regulated by miR-1184 by loading to the COL1A1 3' UTR, we explored the site of COL1A1 that acts on the miR-1184 cluster. Wild-type and mutant sites of the COL1A1 mRNA 3' UTR from positions 1192 to 1198 (COL1A1 wt 3' UTR and COL1A1 mut 3' UTR, respectively) were cloned into the region immediately downstream of the luciferase reporter gene. Next, miR-1184 mimic or inhibitor oligonucleotides were cotransfected with the different luciferase 3' UTR constructs into HG-stimulated HPMCs. miR-1184 decreased the luciferase activity of the wild-type COL1A1 3' UTR. However, the luciferase activity of the mutant COL1A1 3' UTR was unchanged. Therefore, the inactivation of COL1A1 by miR-1184 depends on the COL1A1 3' UTR. We further transfected HG-stimulated HPMCs with COL1A1 plasmids containing the wild-type 3' UTR or lacking the 3' UTR. In HG-stimulated HPMCs transfected with miR-1184, COL1A1 expression was markedly reduced in cells transfected with the wild-type 3'-UTR but not in those transfected with the construct lacking the 3' UTR.

### Downregulation of lncRNA RPL29P2 reversed phenotypic transition by targeting miR-1184-COL1A1 *in vitro*

**Figure 4:**
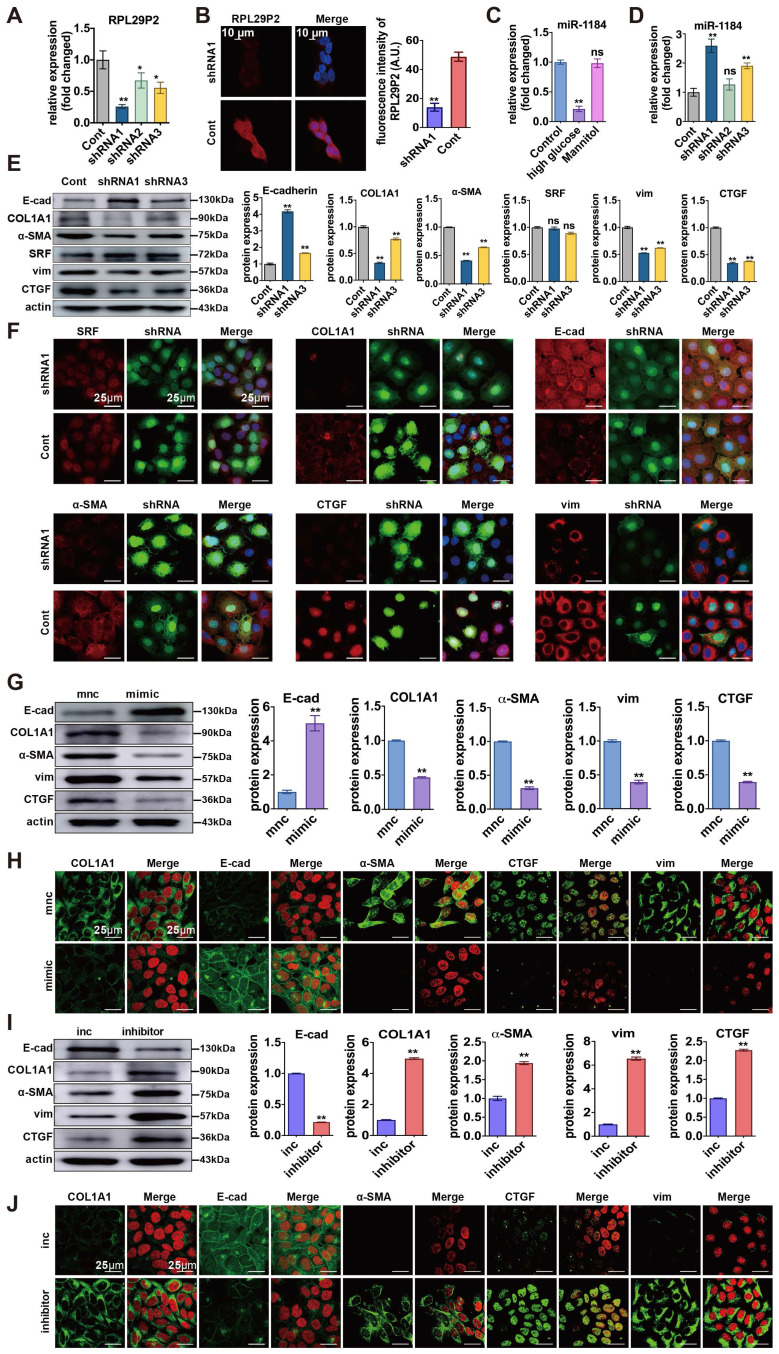
**Downregulation of RPL29P2 reversed the HG-induced phenotypic transition of HPMCs by targeting miR-1184-COL1A1 *in vitro*. (A, B)** PCR and FISH showed the expression of RPL29P2 in HG-HPMGs transfected with shRNA1. **(C, D)** PCR showed the expression of miR-1184 in HG-stimulated HPMCs and transfected HPMCs. *ns*, not significant; ** *P*<0.01. **(E, F)** WB and IF staining showed the expression of SRF, COL1A1, E-cadherin, α-SMA, CTGF, and vimentin after transfection with RPL29P2 shRNA1 lentivirus compared to the control. *ns*, not significant; * *P*<0.05; ** *P*<0.01. **(G, H)** WB and IH staining showed the protein levels of E-cadherin, COL1A1, α-SMA, CTGF, and vimentin in HG-stimulated HPMCs transfected with the miR-1184 mimic. β-Actin was used as the loading control. **P<0.01. **(I)** WB and IH analysis showed the expression of COL1A1, E-cadherin, α-SMA, CTGF, and vimentin in HG-stimulated HPMCs transfected with the miR-1184 inhibitor compared to the control. *β-Actin* was used as the loading control. **P<0.01.

We designed three small hairpin RNAs (shRNA) sequences (shRNA1, shRNA2, and shRNA3). The shRNA1 sequence was used to construct the inhibitor lentivirus, and shRNA3 was used as a control. (Fig. 4A, Table S4A). FISH showed that RPL29P2 was expressed primarily in the cytoplasm and nucleus of HPMCs after stimulation. In HG-stimulated HPMCs transfected with shRNA1 lentivirus, RPL29P2 expression decreased significantly compared to that in the control cells. In contrast, the expression of miR-1184 was upregulated (Fig. 4B-D). The PCR, WB and IH assays showed that in HG-stimulated HPMCs transfected with shRNA1 lentivirus, E-cad was overexpressed, but α-SMA, vimentin and CTGF showed lowered expression levels than in the control HPMCs (Fig. 4E- F, Fig. S3A, Fig. S4C).

We further analyzed miR-1184 and COL1A1 during the reversal of the phenotypic transition and fibrosis in immortalized HPMCs transfected with RPL29P2 shRNA lentivirus. First, PCR showed that COL1A1 was significantly downregulated after transfection with RPL29P2 shRNA1 lentivirus relative to the control (Fig. S3A). However, SRF expression in HG-stimulated HPMCs was unchanged after transfection. WB analysis of SRF and COL1A1 expression showed the same results as qPCR (Fig. S3, Fig. S4). IH showed that the level of COL1A1 was lower in HG-stimulated HPMCs transfected with RPL29P2 shRNA1; however, the expression of SRF showed little change (Fig. 4F, Table S4).

Furthermore, miR-1184 was found to be upregulated after transfection with the miR-1184 mimic compared to control cells (Fig. 4G, H, Fig. S3, Fig. S4). In addition, in HPMCs, the E-cad level significantly decreased, but COL1A1, α-SMA, CTGF, and vimentin increased significantly with the miR-1184 inhibitor compared to the control cells (Fig. 4I, J). The results also showed that in HG-stimulated HPMCs, E-cad was expressed at lower levels, but COL1A1, α-SMA, CTGF, and vimentin was overexpressed with the miR-1184 inhibitor.

### Downregulation of RPL29P2 reversed peritoneal fibrosis and prevented PTF in PD rats

**Figure 5:**
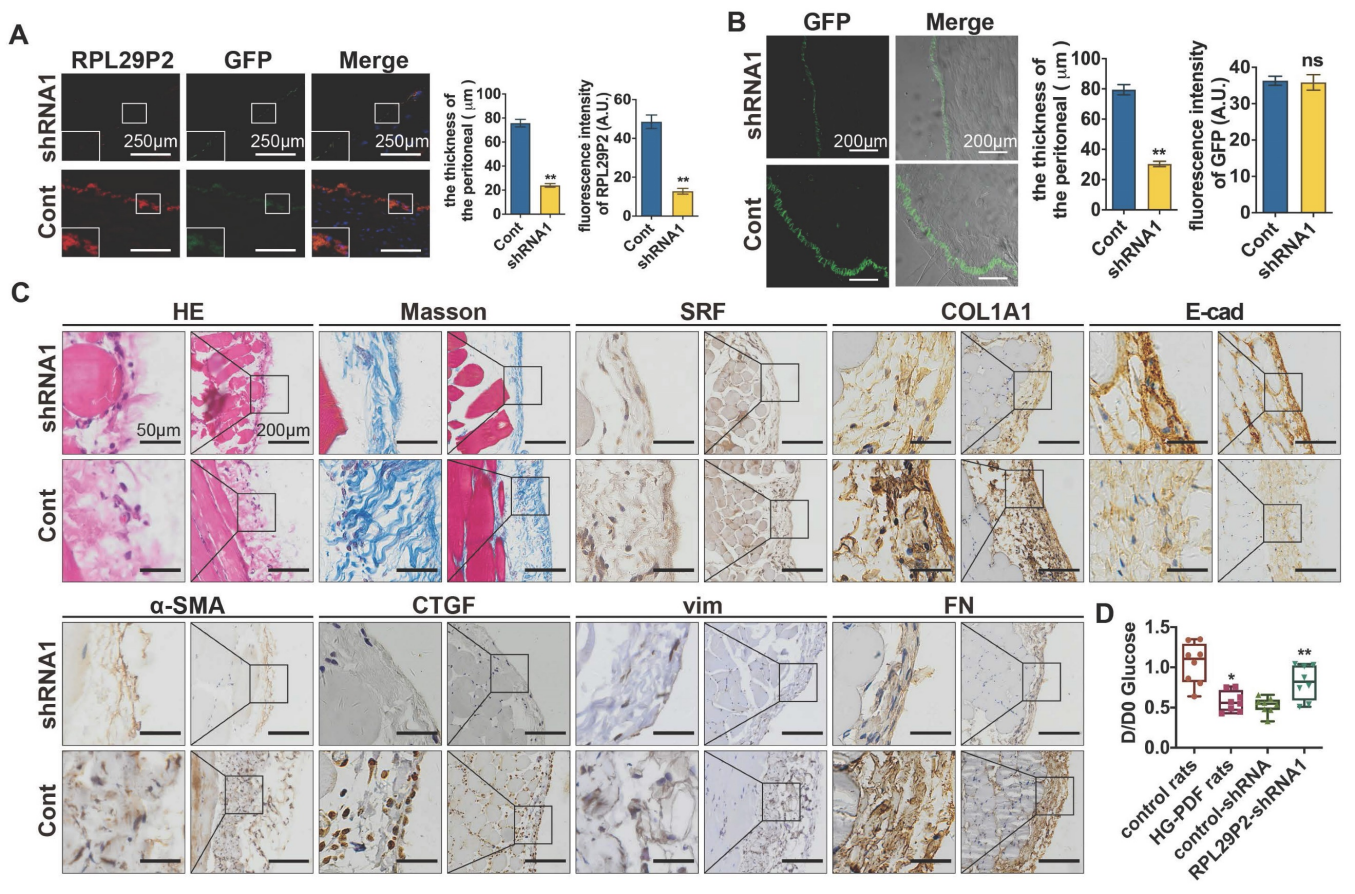
**Downregulation of RPL29P2 reversed peritoneal fibrosis and prevented PTF in PD rats *in vivo* (A)** FISH showed that the expression of RPL29P2 in HG-PDF rats injected with RPL29P2 shRNA1 AAV (shRNA1) compared to control *in vivo*. ** *P*<0.01. **(B)** The peritoneal thickness of rats in the shRNA1 group of HG-PDF rats compared to the control. ** *P*<0.01. **(C)** H&E Masson and IH staining showed the expression of SRF, COL1A1, E-cadherin, α-SMA, CTGF, vimentin and FN in HG-PDF rats treated with RPL29P2 shRNA1 AAV (shRNA1) compared to the control *in vivo*.** (D)** The initial dialysate-to-end dialysate ratio of glucose (D/D_0_ glucose) was used to evaluate the transport status of PM in control rats, HG-PDF rats, and HG-PDF rats injected with control or RPL29P2-shRNA1 adeno-associated virus. *P<0.05 versus control rats; **P<0.05 versus HG-PDF rats injected with control lentivirus.

RPL29P2 shRNA1 AAV was intraperitoneally injected into PD rats (8 rats per group) (Fig. 5). The FISH results showed that RPL29P2 was significantly expressed in PD model rats injected with control vector but was significantly downregulated in AAV-PD model rats infected with AAV-RPL29P2 shRNA1. In PD rats, a thinner PM (23.95±2.62 μm) was measured in the parietal peritoneum after AAV-RPL29P2 shRNA1 treatment than in control rats, which had a PM thickness of 75.73±5.37 μm (Fig. 5A, Table S2E). The results showed a significantly thinner PM in AAV-PD rats (30.34±3.06 μm) than in control PD rats (79.47±5.85 μm) (Fig. 5B, Table S2F). This result indicated that the injection of AAV-RPL29P2 shRNA1 in PD rats results in the thinner PM seen in PD rats.

The PM of PD rats injected with AAV-RPL29P2 shRNA1 exhibited significant downregulation of α-SMA, COL1A1 and FN but an overexpression of E-cad (Fig. 5C) was noticed.

A peritoneal equilibration test was used to test the transfer function of PM., D/D_0_ glucose was measured at 240 min (Fig. 5 D). D/D_0_ in normal control, HG-PDF, control-shRNA and RPL29P2-shRNA rats were 1.045±0.25713, 0.5788±0.12665, 0.5275±0.10138 and 0.8138±0.21186, respectively. This result implied a significant decrease in D/D_0_ glucose in HG-PDF rats and an increase in RPL29P2-shRNA1 compared with control-shRNA rats.

### RPL29P2 was positively correlated with SRF and COL1A1 but negatively correlated with miR-1184 in ex vivo primary HPMCs from continuous ambulatory PD (CAPD) patients

**Figure 6:**
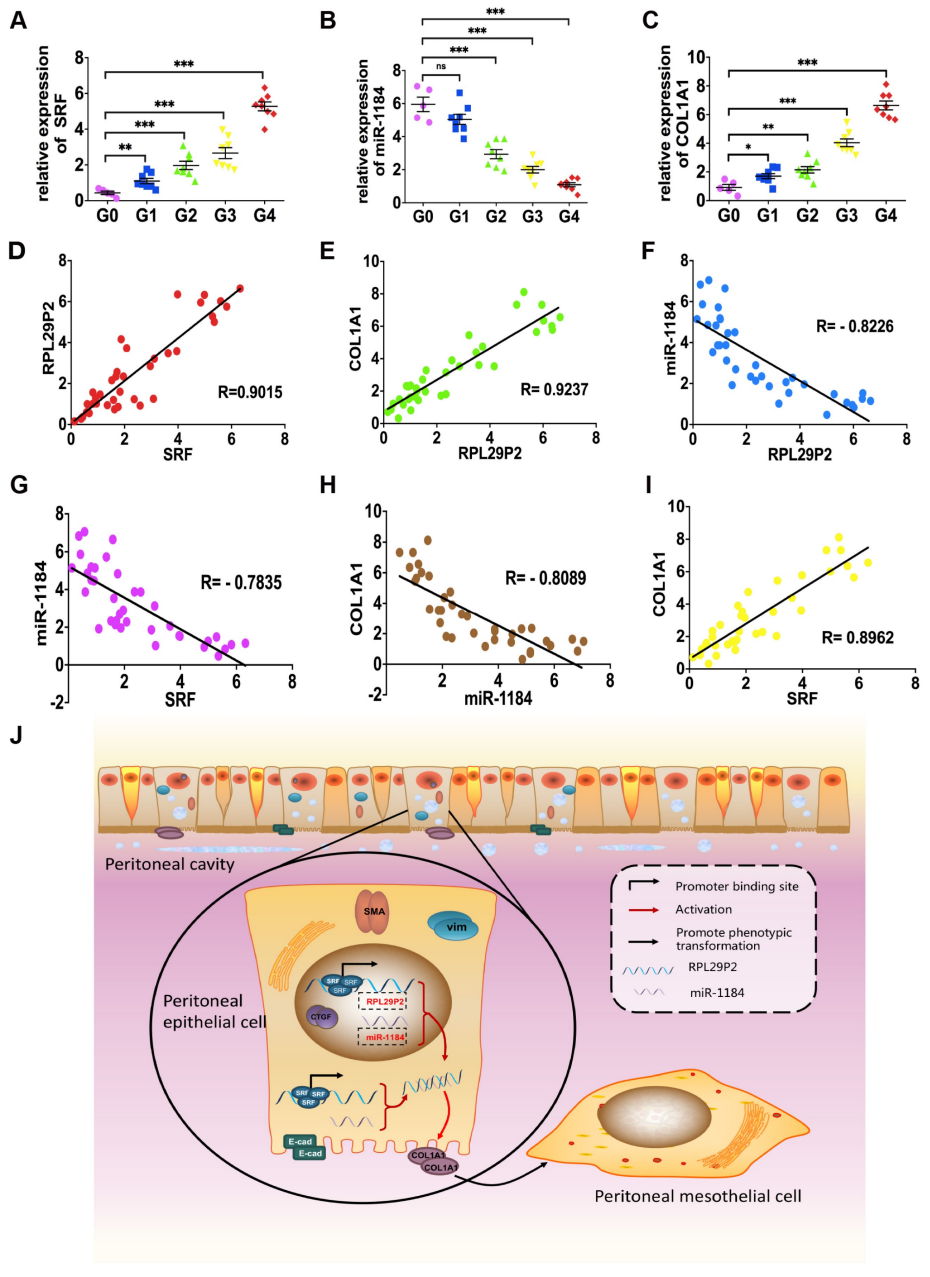
** RPL29P2 was positively expressed with COL1A1 but negatively expressed with miR-1184 ex vivo. (A-C)** PCR showed the expression of SRF, miR-1184, and COL1A1 in effluent-derived HPMCs from 32 PD patients (G1-G4) who underwent PD treatment and in omentum-derived HPMCs from five patients with ESRD who underwent catheterization surgery for further PD therapy as a control group (G0). **(D-I)** Correlation analysis showed correlations between SRF and RPL29P2, RPL29P2 and COL1A1, SRF and COL1A1, SRF and miR-1184, miR-1184 and COL1A1, and miR-1184 and RPL29P2 in effluent-derived HPMCs from groups G0 to G4. R Sq Line ar, R-squared line-adjusted rate. **(J)** Schematic summarizing the SRF-RPL29P2-miR-1184-COL1A1 axis in HG-stimulated HPMCs during peritoneal fibrosis. Upon exposure to HG-PDF, HPMCs showed profound phenotypic transition changes, including the gain of mesenchymal characteristics and transition to a fibroblast-like morphology. Active SRF was translocated from the cytoplasm into the nuclei of HPMCs, where it bound to the CArG element and promoted the expression of the RPL29P2 gene. Increased RPL29P2 bound to miR-1184 through sponge adsorption, thus reducing the expression of miR-1184, which can bind to COL1A1 mRNA, resulting in the loss of the epithelial junction protein E-cadherin but an increase in collagen deposition. Therefore, the HG-induced phenotypic transition of HPMCs may facilitate their migration into the submesothelial zone through the disrupted PM and contribute to the increased deposition of extracellular matrix, ultimately leading to PMF.

Omentum-derived HPMCs from 5 ESRD patients underwent catheterization surgery, and HPMCs from 32 PD dedicators were collected (Fig. 6, Table. S3).

The levels of SRF, RPL29P2 and COL1A1 were significantly higher in HPMCs in G2, G3 and G4 CAPD dedicators than in those from G0 patients (Fig. 6A, C, Fig. 1D, Table. S3). However, we observed lower expression of miR-1184 in G1, G2, G3 and G4 patients who underwent more than one year of treatment than in G0 patients who underwent short-term CAPD (Fig. 6B). RPL29P2 was positively correlated with SRF and COL1A1 but negatively correlated with miR-1184 (Fig. 6D-F). Additionally, miR-1184 gene expression was negatively correlated with SRF and COL1A1 expression (Fig. 6G, H), and SRF expression was positively correlated with COL1A1 expression (Fig. 6I). RPL29P2 gene expression was positively correlated with D/P but negatively correlated with D/D_0_ in the PD patients (Fig. 1D). Thus, RPL29P2 may be associated with the phenotypic transition of HPMCs following injury of the PM and attenuation of transport function by HG exposure during long-term PD.

## Discussion

Peritoneal fibrosis (PF) is a serious complication that may fail the PD success in uremic patients 24, 25. A key point in clinical therapy is to avoid or inhibit the PF progression. Among the various factors with adverse effects on the PM, such as uremia, extended PD duration, peritonitis, glucose, and glucose degradation products, HG is one of the primary factors consistently focused on in our study 26, 27. Previous work had shown that HG-stimulated EMT of HPMCs is the earliest reversible change during the initial period 28. Mesothelial cells are the main source of extracellular matrix components that increase the expression of COL1A1 and produce collagens, leading to PMF 29, 30. Thus, the earliest reduction or inhibition of the progression of the phenotypic transition of mesothelial cells might prevent PF during PD 31.

SRF directly promoted PF via the same miRNAs or miRNA clusters, such as the miR-199a/214 cluster identified in previous studies 10. These findings showed that hundreds of CArG-containing boxes in miRNAs might be targets of SRF. However, these target miRNAs did not contain CArG boxes in their promoter regions. Other factors might participate in the HG-induced phenotypic transition of HPMCs as intermediaries between transcription factors, miRNAs, and these target genes. LncRNAs might be important factors that could be regulated by transcription factors and subsequently sponge miRNAs, thus affecting the downstream target genes that mediate PF. LncRNAs have many biological effects and their abnormal expressions are associated with tumor metastasis and metabolic, cell proliferation, and fibrotic diseases 31-34. Recent studies have shown that lncRNAs could significantly regulate PF 35-38. Unlike miRNAs and small nucleolar RNAs, lncRNAs often lack high conservation in their functional region. More work is needed to study the role of lncRNAs in fibrosis during PD treatment.

Our study provides emerging evidence that human lncRNAs, such as RPL29P2, which are regulated by SRF, can regulate cellular phenotypic transitions by sponging miRNAs, such as miR-1184 and its target gene COL1A1. Initially, because a single transcription factor can regulate multiple lncRNAs and the corresponding downstream miRNAs, we designed a screen of an array of lncRNAs in HPMCs and RPMCs to identify differentially expressed lncRNAs during the HG-induced phenotypic transition in peritoneal fibrosis. Our results showed that RPL29P2 was one of the most differentially expressed factors that might contribute to fibrogenesis-related signaling pathways during PD treatment. More interestingly, RPL29P2 is only one of the target lncRNAs that can be directly regulated by the transcription factor SRF, a promoter of peritoneal fibrosis. SRF-lncRNA RPL29P2 pathway also could presumably enhance peritoneal fibrosis by sponging downstream miRNAs and their target genes or target proteins. By biological and sequence analysis, we found that miR-1184 might be one of the downstream miRNAs involved in this process. The results of reporter assays and cell-based experiments proved our hypothesis. Finally, our results showed a complete pathway by which the transcription factor SRF could enhance HG-induced peritoneal fibrosis during PD; one pathway functions through direct upregulation of lncRNA RPL29P2 expression. The lncRNA RPL29P2 could promote phenotypic transition by sponging miR-1184. One predicted target of miR-1184 is COL1A1. Under normal conditions, COL1A1 mRNA could be sponged by miR-1184 to prevent additional collagen deposition and peritoneal fibrosis. In contrast, in HG-stimulated HPMCs, high expression of SRF-lncRNA RPL29P2 led to the inhibition of miR-1184 and alleviated the sponge effect on COL1A1 mRNA, causing increased collagen and ECM deposition and ultimately enhancing PF (Fig. 6J).

In recent years, a large number of studies have explored the relationship between lncRNA and kidney disease. There is emerging evidence for the involvement of lncRNAs in many kidney diseases, including acute kidney injure (AKI), chronic kidney disease (CKD), diabetic nephropathy 16, 35, renal fibrosis and so on. Many studies shown that lncRNAs are involved in the development of septic AKI and in turn resulted in a severe inflammatory response 39. A series of lncRNAs suggested regulate critical cell cycle regulators, such as the cyclins, CDKs, and p53, thereby affecting cell proliferation and tissue repair. In addition, some lncRNAs are upregulated in renal fibrosis and play a pro-fibrotic role 40-42. In research related to kidney transplantation, it has been found that lncRNAs are also involved in TGF-β/Smad3 pathway, and up-regulated of certain lncRNAs could also act as a novel biomarker of acute kidney rejection 43. But there is few researches on lncRNA in PD-associated PF. In this study, we found that a new lncRNA, RPL29P2, was upregulated in the peritoneal membrane of long-term PD patients, and its expression level was correlated with peritoneal fibrosis severity. Our research confirms that lncRNA RPL29P2 promotes PF and impairs peritoneal transport function via miR-1184 and it will provide new ideas for the treatment of PF.

Our study may be limited by some aspects. First, HPMCs obtained from the PD effluent of patients may mainly constitute the cells that fall off the peritoneum because of the phenotypic transition of HPMCs. The PM is made up of various kinds of cells; thus, the sloughed cells could not completely represent the HPMCs in the actual human PM. Second, RPL29P2 was firstly selected from rats. The selected results from immortalized HPMCs showed that RPL29P2 was not the most altered factor after HG stimulation. However, the PCR results showed the differential expression of RPL29P2 after treatment with HG. The selected array might also have its own limitations. The results only showed that RPL29P2 is a differentially expression factor in HPMCs after HG stimulation, but its expression could also be regulated by SRF in these cells. Thus, other pathways require additional experiments and further research. Thirdly, we designed an experiment to silence the RPL29P2 in the rat model for therapeutic purposes. However, unlike other ncRNAs, such as miRNAs, which are highly conserved between different species, lncRNAs generally lack conservation between species 44. Therefore, the question you raised is one of the limitations of our research, which is that the results on animal models may not be entirely applicable to human patients. Meanwhile, potential off-target effects cannot be completely avoided.

## Supplementary Material

Supplementary figures and tables.
