# Supplementary figures and images for: LncRNA RPL29P2 promotes peritoneal fibrosis and impairs peritoneal transport function via miR-1184 in peritoneal dialysis
Source: Int J Med Sci. 2024 Apr 15;21(6):1049–63. doi: 10.7150/ijms.93547 (PMC11103403; doi:10.7150/ijms.93547)

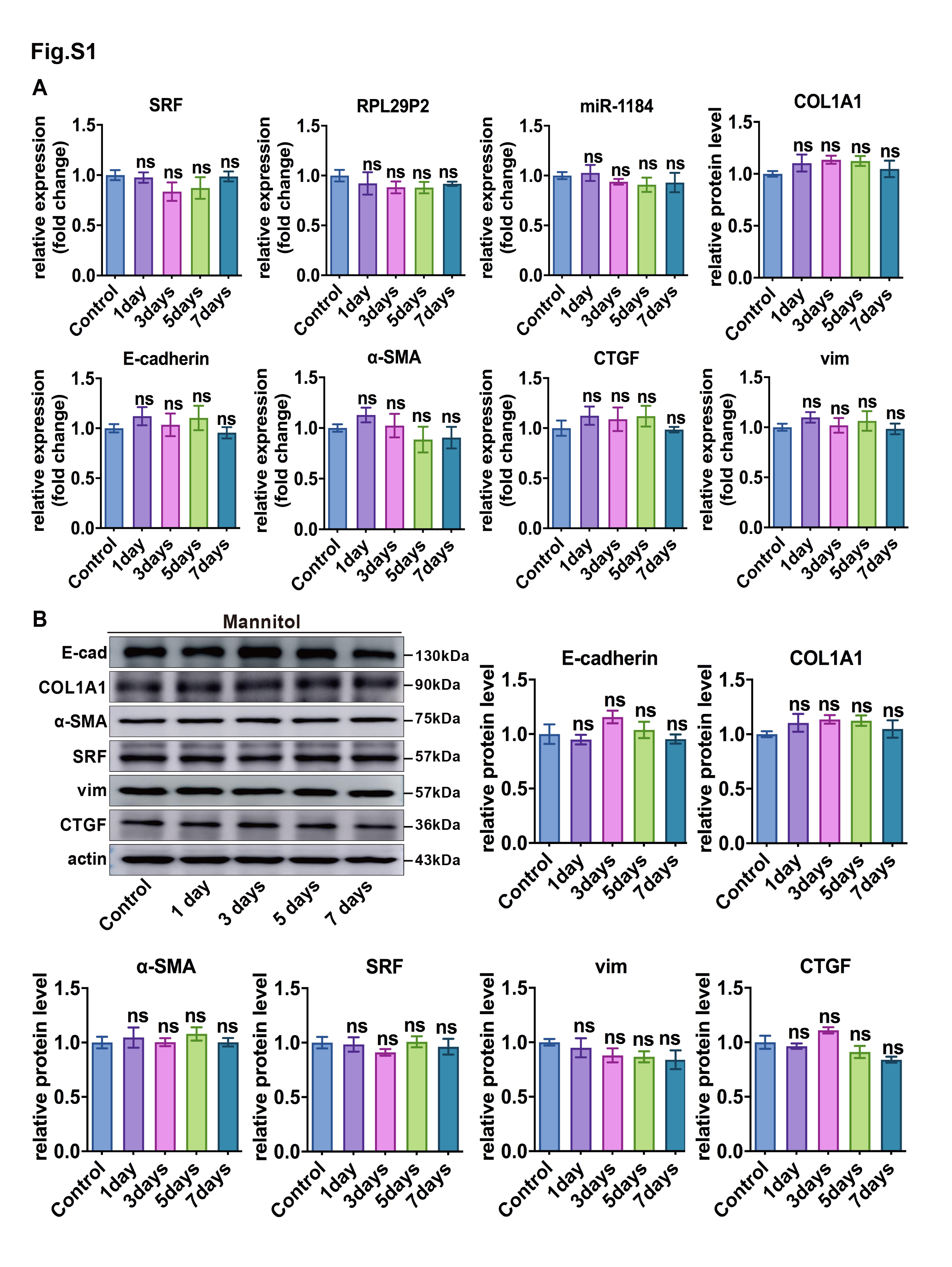

Supplement: Supplementary file 1 — Supplementary figures and tables. [file ijmsv21p1049s1.zip › Supplementary Materials/Fig. S1.tif]

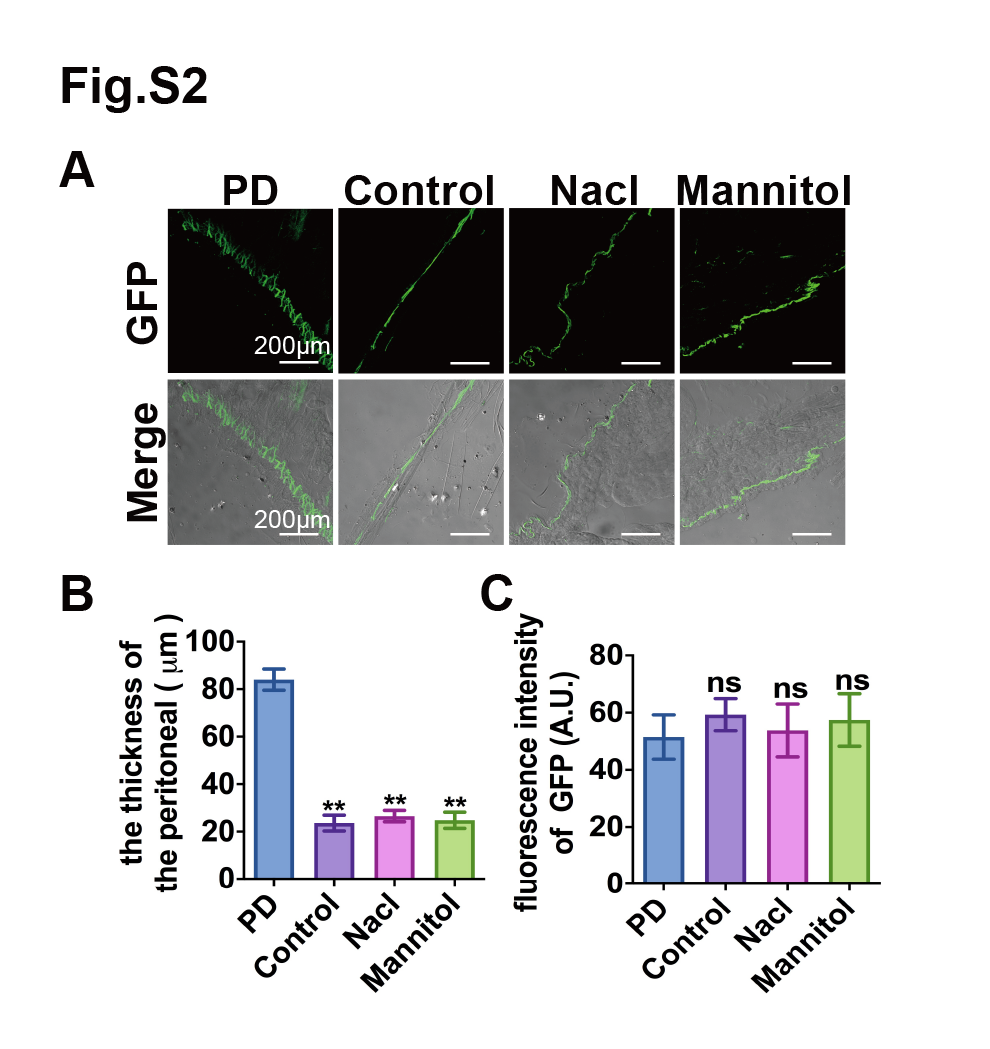

Supplement: Supplementary file 1 — Supplementary figures and tables. [file ijmsv21p1049s1.zip › Supplementary Materials/Fig.S2.tif]

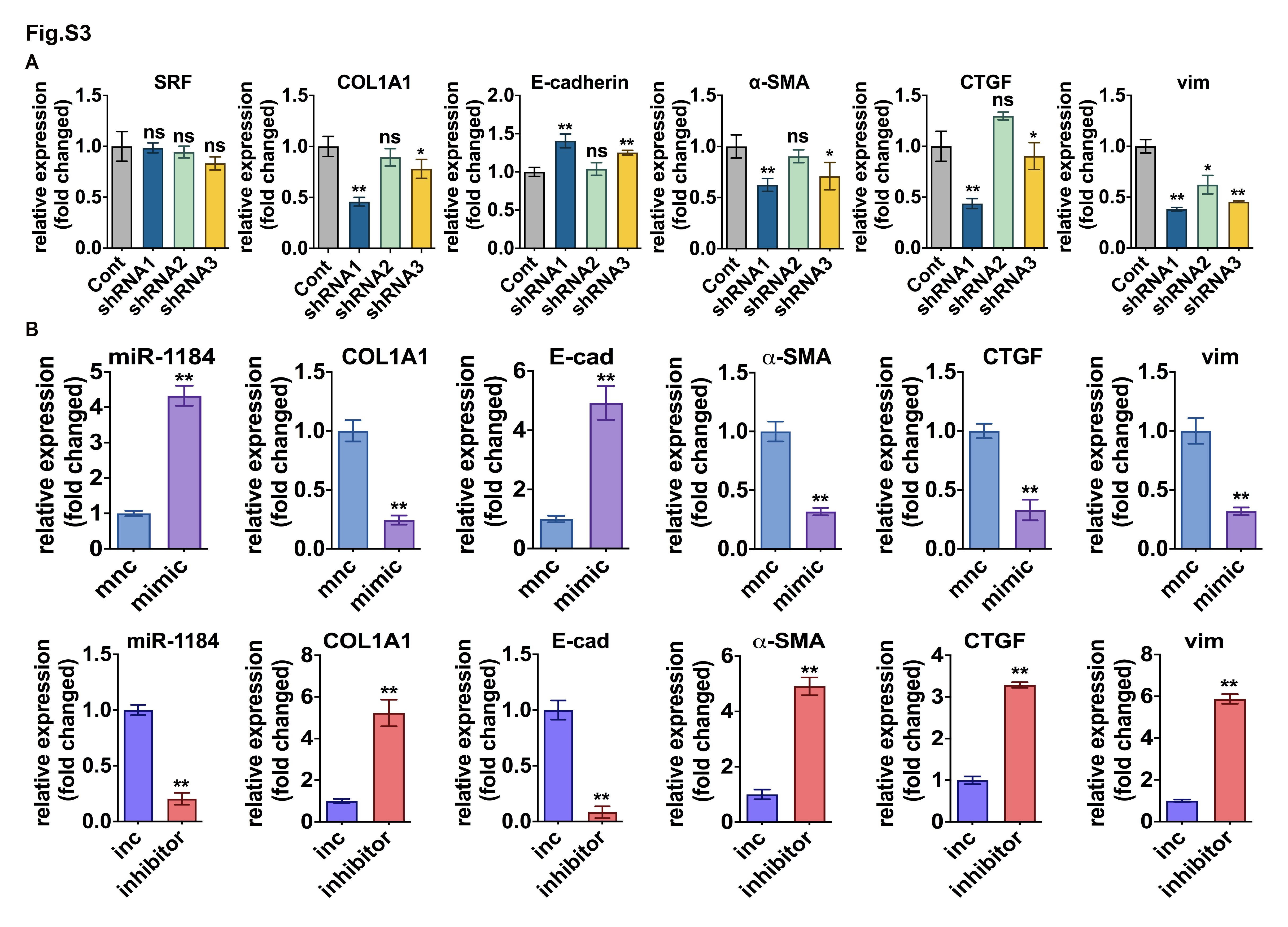

Supplement: Supplementary file 1 — Supplementary figures and tables. [file ijmsv21p1049s1.zip › Supplementary Materials/Fig.S3.tif]

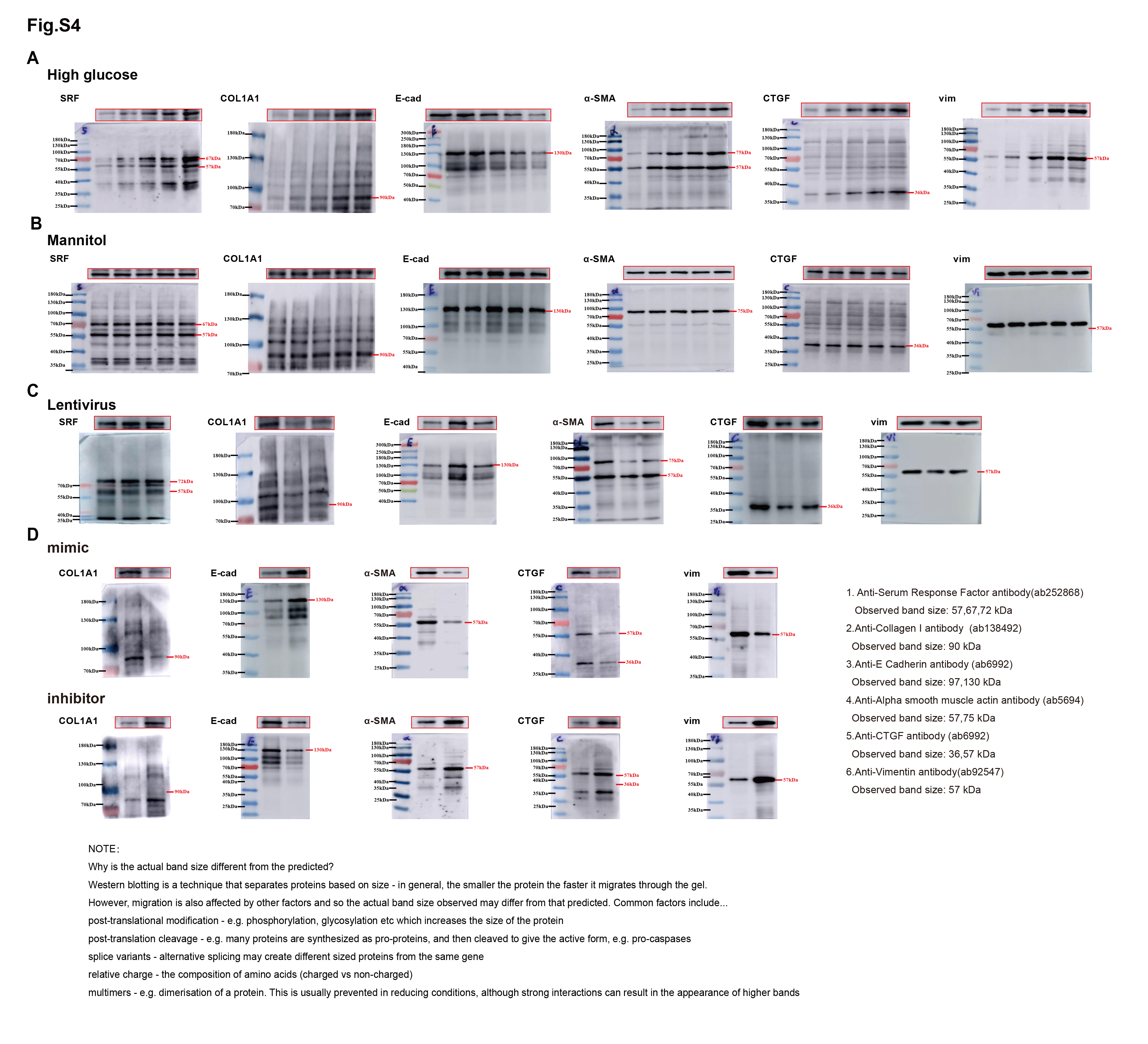

Supplement: Supplementary file 1 — Supplementary figures and tables. [file ijmsv21p1049s1.zip › Supplementary Materials/Fig.S4.tif]
